# Perceptions, behaviours and attitudes towards smoking held by the male partners of Chinese pregnant women: a qualitative study

**DOI:** 10.1186/s12889-021-11966-4

**Published:** 2021-10-20

**Authors:** Wei Xia, Ho Cheung William Li, Peige Song, Ka Yan Ho, Yuanhui Luo, Tingna Liang, Laurie Long Kwan Ho, Ankie Tan Cheung, Wenzhi Cai

**Affiliations:** 1 School of Nursing, Sun Yat-Sen University, Guangzhou, China; 2 School of Nursing, The University of Hong Kong, Hong Kong, SAR China; 3 The Nethersole School of Nursing, The Chinese University of Hong Kong, Shatin, New Territories, Hong Kong; 4 School of Public Health, Zhejiang University School of Medicine, Hangzhou, China; 5 Centre for Global Health Research, Usher Institute of Population Health Sciences and Informatics, The University of Edinburgh, Edinburgh, UK; 6 School of Nursing, The Hong Kong Polytechnic University, Hong Kong, SAR China; 7 Shenzhen hospital, Southern Medical University, Shenzhen, China

**Keywords:** Chinese expectant fathers, Pregnant women, Smoking cessation, Smoking behaviour, Qualitative study

## Abstract

**Background:**

Direct associations of tobacco exposure during pregnancy with pregnancy complications and adverse birth outcomes have been proven. Previous studies suggest that expecting a child provides a valuable opportunity to promote behavioural changes, such as smoking cessation, among the male partners of pregnant women. Thorough understandings of Chinese expectant fathers’ smoking behaviour during the transition to fatherhood is a prerequisite to the development of appropriate interventions to facilitate smoking cessation. This study aimed to explore the perceptions, behaviours and attitudes related to smoking among male partners of pregnant women in China.

**Methods:**

A descriptive phenomenological approach was adopted. A purposive sample of expectant fathers aged 18 years or older who had a tobacco use history within the past year were recruited at obstetrics and gynaecology clinics and invited to participate in one-to-one, 20–30-min semi-structured interviews. The data analysis followed Colaizzi’s descriptive phenomenological method.

**Results:**

Twenty-five expectant fathers were interviewed. Four themes were generated: 1) the benefits of smoking and respondents’ misperceptions of the impact of smoking and SHS and neglectful attitude of the impact of smoking, which were given as the major reasons for continuing to smoke; 2) factors contributing to smoking cessation, including concern for the potential health impact of continued smoking on the pregnant partner and baby, the role of being father, and the encouragement to quit from family members; and 3) perceived barriers to smoking cessation, including withdrawal symptoms or cigarette cravings, absence of smoking cessation support, and increasing stress.

**Conclusion:**

This study provides a comprehensive understanding of the perception, behaviours, and attitudes related to smoking among Chinese expectant fathers. The findings of this study can guide healthcare professionals and policymakers in combining the distribution of educational information about the hazards of SHS for maternal and neonatal health with smoking cessation assistance for expectant fathers through policy initiatives and other types of incentives and programmes targeted to enhance smoking cessation among this population.

**Trial registration:**

Prospectively registered at clinicaltrial.org (NCT03401021) on 8 Jan 2018.

**Supplementary Information:**

The online version contains supplementary material available at 10.1186/s12889-021-11966-4.

## Background

Research has proven a direct link between exposure to second-hand smoke (SHS) during pregnancy and pregnancy complications and adverse birth outcomes, including preterm delivery, spontaneous abortion, low birth weight and even foetal death [1, 2]. China is the largest consumer of tobacco worldwide, with more than 300 million current smokers, of which more than 95% are men [3]. In previous studies, only 3.8% of Chinese pregnant women smoked, whereas approximately 42.9% of their male partners were smokers before the pregnancies [4, 5]. Chinese smoking culture has a long and influential history, and smoking serves a particularly important social function in terms of forging connections between individuals in China [6]. Given this cultural benefit of smoking during daily interactions, smoking by Chinese men is well tolerated by others around them, including their pregnant wives [7, 8]. Approximately 75% of Chinese non-smoking pregnant women with a smoking partner reported that they were primarily exposed to SHS from their smoking partners during their pregnancy [9]. An investigation found that only 6.5% of Chinese smokers who became expectant fathers quit smoking during their partners’ pregnancies, and most relapsed after their wives had delivered the babies [10]. Therefore, it is crucial to help the partners of pregnant women to quit smoking, with the aim of protecting smokers, pregnant women and their new-borns from the hazards of smoking and SHS exposure.

An expectant father presents a golden opportunity for promoting health behaviours, as the changes inherent to the new role may increase a man’s receptivity to health-related behavioural information and interventions [11]. In a systematic review, the data synthesis revealed that men were often stimulated to attempt smoking cessation during their partners’ pregnancies and after delivery [12]. A review of the literature reveals a few studies in Western countries, including the US, that were targeted at helping expectant fathers with smoking cessation [12, 13]. However, the results of these studies [12, 13] were unpromising. A study conducted in the US identified that expectant fathers’ current smoking was associated with having a lower level of education, a pregnant partner who was a current smoker, and the absence of smoking prohibitions inside the home [14]. While the study identified knowledge about the health hazards of SHS exposure for pregnant women, foetuses and new-borns, being a first-time expectant father and having good family support were factors associated with successful abstinence from tobacco use among Chinese expectant fathers [15]. A thorough understanding of the perceptions, behaviours and attitudes related to smoking among Chinese expectant fathers during the transition to fatherhood is a prerequisite to the development of appropriate interventions to facilitate smoking cessation. However, the investigations above neither provided important information explaining how the identified factors affected smoking cessation, nor yielded insights into the factors that facilitate or discourage successful abstinence based on the experiences of expectant fathers who had quit or who had continued smoking [15]. In a review of fathers’ views on smoking cessation during their partners’ pregnancy, all identified studies were conducted in Western countries [16], and this made it difficult to apply the results to Chinese expectant fathers who smoke. Therefore, this study aimed to explore the perceptions, behaviours and attitudes related to smoking among Chinese expectant fathers; it particularly aimed to identify any barriers or facilitators to smoking cessation during the transition to fatherhood.

## Methods

### Study design

This qualitative study applied a descriptive phenomenological approach involving a series of individual semi-structured interviews, with the aim of producing a comprehensive description of a phenomenon related to the lived experiences of expectant fathers with respect to smoking [17, 18]. We also aimed to identify any barriers and facilitators to smoking cessation among Chinese expectant fathers who smoked during their transition to fatherhood. This study was reported according to the Consolidated Criteria for Reporting Qualitative Research (COREQ), and a COREQ checklist was completed [19].

### Study population

The samples were selected from a cohort of smoking male partners of pregnant women at the obstetrics and gynaecology clinics of three tertiary hospitals in China [15]. To fully understand the male partners’ perceptions, behaviours and attitudes related to smoking and strengthen the transferability of the findings, purposive sampling was performed according to the men’s smoking cessation trajectories before and after their partners became pregnant and based on related characteristics collected in a previous survey to maximise the sample variation and representation [20]. The sample size was determined by data saturation at which point no new information was extracted from more than three consecutive informants [21].

### Research team

A research panel was constructed (Supplementary 1) to include two associate professors, one assistant professor, one research professor, three post-doctoral fellows and one PhD candidate. All the members had at least three years of experience in conducting smoking cessation and qualitative research. In the previous cross-sectional survey, the members of the research panel had established good relationships with the participants, which enabled them to complete high-quality interviews.

### Interview guide

A semi-structured interview template was used to guide the interviews (Supplementary 2). Each conversation was initiated by an inquiry about changes in tobacco use from before to after the interviewee’s partner became pregnant, after which the interviewee was asked about his experience with and opinions about smoking and cessation. The interview guide was examined to ensure the relevancy and appropriateness of the wording.

### Data collection

Eligible expectant fathers were approached via telephone and invited to complete a face-to-face, individual semi-structured interview. All interviews were conducted in Chinese by the same two facilitators according to the semi-structured interview guide and were digitally recorded. An independent researcher observed the interviews and documented the interviewees’ non-verbal cues as triangulation to enhance the validity of this study. To ensure data credibility, the interviewers were requested to use probes and iterative questions during the interview to ensure the credibility of the data [22]. All interview recordings were transcribed verbatim in Chinese as soon as possible after the interview to capture the nuances of expression unique to the dialect and identify the relevant quotations. To ensure the appropriateness of the interview guide, we modified and added more specific questions according to the summarised ideas and interpretations from debriefing sessions and discussed the saturation after every five interviews [23].

### Data analysis

QSR NVivo software version 12 was used to store and analyse the qualitative data according to Colaizzi’s (1978) method of descriptive phenomenological data analysis [24, 25]. The textual units, which comprised the individual words, phrases and sentences in the scripts, were examined line-by-line. Initially, open-coding was conducted by two independent researchers while considering the field notes. The research team met to discuss the coding after the first five interviews were processed. Areas of divergence were settled through discussion until consensus was reached or the materials were sent to the interviewees for review or clarification. To ensure consistency, the smoking behaviours described by interviewees during the interviews were also compared with their assessment records, and the participants verified that the descriptions reflected their experiences. After a systematically parallel comparison to ensure mutual exclusion, the code components were synthesised and are presented with illustrations of quotations from the transcripts and have been translated into English. To ensure the confirmability and dependability of the findings, an experienced bilingual researcher who was not involved in this study audited the documented interpretations to eliminate bias that might have arisen from preconceived ideas and ensure the validity of the results. The ‘thick description’ strategy was used to ensure the transferability of the data. Explicit descriptions of the research design and methods and examples of raw data were provided to ensure that the conclusions made in the study are transferable to other settings with similar participants and contexts.

## Results

From February to March 2018, 32 eligible smoking expectant fathers were approached. Of them, 7 refused to participate: 2 reported a lack of time and 5 reported a lack of interest. Finally, 25 expectant fathers participated in the interviews and data saturation was achieved with no dropouts. The interviewees’ demographic and smoking characteristics are presented individually in Table 1 and summarised in Table 2. The interviewees’ ages ranged from 22 to 46 (mean 33.7, SD 5.8) years. After their partners became pregnant, 5 interviewees (20.0%) quit smoking, 7 (28.0%) quit but relapsed, 4 (16.0%) attempted to quit but failed and 9 (36.0%) never tried to quit smoking. Seventeen interviewees (68%) were employed, 19 (76%) had an education level of college or higher, 17 (68.0%) had an annual income of 50,000–199,999 CNY (equal to 7453.7-29,814.6 USD), 14 (56.0%) had experienced stressful events within the last 30 days, 16 (64.0%) were expectant fathers for the first time and 14 (60.9%) had attended pregnancy education. The participants’ mean daily cigarette consumption was 11.1 (SD 9.1). Sixteen interviewees (64.0%) had attempted to quit within the past year, and 11 (44.0%) were still in the pre-contemplation stage with no intention to quit smoking in the next 6 months.

**Table 1:** Participants’ individual demographic characteristics and smoking profiles (*n* = 25)

Informant ID	Age rang*, years	Smoking status ^a^	Stage of readiness to quit ^b^	Employment status ^c^	Education level	Annual income ^d^	New father	Stress event within 30 days	Attendance of pregnancy education
001	18–35	F	C	Un-E	Secondary	Level 3	Yes	Yes	Yes
002	18–35	R	P	E	≥ College	Level 3	Yes	No	No
003	18–35	S	Pre-C	Self-E	≥ College	Level 3	No	Yes	No
004	36–55	R	A	Un-E	Secondary	Level 4	Yes	No	Yes
005	18–35	S	Pre-C	E	≥ College	Level 3	Yes	Yes	Yes
006	18–35	F	P	Un-E	≥ College	Level 3	Yes	No	Yes
007	36–55	R	A	Self-E	≥ College	Level 1	No	No	No
008	36–55	S	C	E	≥ College	Level 3	No	Yes	No
009	36–55	Q	A	E	≥ College	Level 1	Yes	No	Yes
010	18–35	S	Pre-C	E	Secondary	Level 3	Yes	Yes	No
011	36–55	S	Pre-C	E	≥ College	Level 1	Yes	No	Yes
012	36–55	F	Pre-C	E	Secondary	Level 3	Yes	Yes	Yes
013	18–35	S	Pre-C	E	≥ College	Level 3	Yes	No	Yes
014	36–55	R	C	E	Secondary	Level 2	Yes	Yes	Yes
015	18–35	Q	A	E	≥ College	Level 2	No	Yes	No
016	18–35	F	C	Un-E	≥ College	Level 2	Yes	Yes	No
017	18–35	R	C	E	≥ College	Level 2	No	Yes	Yes
018	18–35	Q	A	E	≥ College	Level 1	No	Yes	Yes
019	18–35	S	Pre-C	Un-E	≥ College	Level 2	Yes	Yes	Yes
020	18–35	R	Pre-C	Self-E	≥ College	Level 4	Yes	Yes	Yes
021	18–35	Q	A	E	Secondary	Level 1	No	No	No
022	18–35	S	Pre-C	E	≥ College	Level 3	No	No	Yes
023	36–55	R	Pre-C	E	≥ College	Level 4	No	No	No
024	18–35	Q	A	E	≥ College	Level 2	Yes	No	No
025	36–55	S	Pre-C	E	≥ College	Level 2	Yes	Yes	No

Note:* To ensure the participant anonymity, the age is reported as age-rangea

^a^: Q = quitter, R = quit but relapsed, F = attempted to quit but failed, S = smoking without quit attempt

^b^: Pre-C = Pre-contemplation stage (no intention to quit within 6-months), C=Contemplation stage (1-month< intend to quit < 6-months), P = Preparation stage (intend to quit within 1-month), A = Action stage (take action to quit)

^c^: Un-E = unemployed, Self-E = self-employed, E = employed

^d^: Level 1 = 49,999 CNY or below, level 2 = 50,000–100,000 CNY, level 3 = 100,000–200,000 CNY, level 4 = over 200,000 CNY. ¥/CNY represents China Yuan, US$1.00 = ¥ 6.7

**Table 2:** Summary of participants’ demographic characteristics and smoking profiles (n = 25)

	n (%)/ mean ± SD
Smoking status	
Quitter	5(20.0)
Quit but relapsed	7(28.0)
Attempted to quit but failed	4(16.0)
Without quit attempts	9(36.0)
Age (years), mean ± SD	33.8 ± 6.0
Employment status	
Employed	17(68.0)
Self-employment	3(12.0)
Unemployed	5(20.0)
Education level	
Middle school	6(24.0)
College/university or above	19(76.0)
Annual family income (CNY) ^a^	
¥ 49,999 or below	5(20.0)
¥ 50,000–99,999	7(28.0)
¥ 100,000–199,999	10(40.0)
¥ 200,000 or above	3(12.0)
Experience stressful event within 30 days	
Yes	14(56.0)
No	11(44.0)
First time as an expectant father	
Yes	16(64.0)
No	9(36.0)
Attendance of pregnancy education	
Yes	14 (56.0)
No	11 (44.0)
Daily cigarette consumption, mean ± SD	11.1 ± 9.1
Level of readiness to quit	
Pre-contemplation (no intention to quit within 6-months)	11(44.0)
Contemplation (1-month< intend to quit < 6-months)	5(20.0)
Preparation (intend to quit within 1-month)	2(8.0)
Action (take action to quit)	7(28.0)

Data are n(%) and mean (SD) unless stated otherwise

^a^ ¥/CNY represents China Yuan, US$1.00 = ¥ 6.7

Four themes were generated and are presented in Table 3.

**Table 3:** Summary of themes and sub-themes from the semi-structured interview

Themes	Sub-themes
Reasons for continuing smoking	Benefits of smoking
	Misperceptions of the impact of smoking and SHS
	The neglectful attitude of the impact of smoking
Factors contributing to smoking cessation	Health concerns on the pregnant partner and baby
	The role of being father
	Encouragement of quitting from family members
Perceived barriers to smoking cessation	Withdrawal symptoms or cigarette cravings
	Absence of smoking cessation support
	Increasing stress

### Theme 1. Reasons for continuing smoking

Most of the participants knew that smoking had a negative impact on the health of their pregnant partners and themselves. However, 20 expectant fathers continued smoking. Three sub-themes were generated to explain the reasons for continuing to smoke, including the benefits of smoking, misconceptions about smoking, and neglectful attitude of the impact of smoking and SHS on health.

#### Benefits of smoking

Most participants, especially those who worked in sales or finance, claimed that because of the influence of the Chinese smoking culture, sharing cigarettes was an efficient method to establish initial relationships with customers. Adult men may be considered impolite if they refuse a shared cigarette and would suffer additional social pressure and even humiliation. The need for social interaction in the workplace was a major factor in smoking behaviour.

*‘When I found a potential customer, sharing cigarettes was an efficient way to close the relationship without being embarrassed. When you smoke together, you can chat by the way, and business may be negotiated. Also, it is difficult to refuse cigarettes offered by others. There is no better way yet. So, it is impossible -- there is no way to quit.’ (Informant 017)*

#### Misperceptions of the impact of smoking and SHS

Misconceptions about the impact of smoking and SHS on health were the main reasons cited by expectant fathers to explain smoking resumption. The expectant fathers held skeptical views of the effect of SHS on the foetus. It was difficult for them to understand how the SHS could negatively influence the health of a foetus that was protected inside the mother. They felt that the negative effects of smoking and SHS on health were exaggerated by healthcare professionals.

*‘I heard that smoking is not good for pregnant woman and foetus, but I cannot understand and see how the foetus could be influenced, the foetus in the mom’s belly and can’t even breathe.’ (Informant 008)*

Those who reported quitting smoking in preparation for pregnancy but later relapsed reported the belief that although smoking may affect the quality of sperm, this effect may not persist after conception.

*‘I stopped smoking when we prepared for the pregnancy, there was no need to stop smoking after my wife got pregnant successfully. And I smoked only in the toilet or when she was not at home. I think it’s enough.’ (Informant 014)*

#### The neglectful attitude of the impact of smoking

Some participants reported that they well knew that smoking was harmful to their health, but they still expressed a neglectful attitude of the negative impact of smoking and stated a willingness to continue smoking. Such neglectful attitude was reported more among the younger men. They explained that that the negative health impacts of smoking developed slowly and would occur in the distant future, when they are old and get sick whether they smoke or not.

*‘Everyone knows smoking can cause diseases, but it is a problem [I will face] decades later!’ (Informant 012)*

*‘Maybe I have dead for other reasons before the negative impact of smoking occurred’ (Informant 003)*

### Theme 2. Factors contributing to smoking cessation

The participants who had quit smoking when their partners got pregnant reported that several factors could facilitate their smoking cessation. According to the interview, three factors were summarized, including concerns about the health impact on the pregnant partner and baby, the role of being a father, and encouragement to quit from family members.

#### Health concerns of the pregnant partner and baby

Expectant fathers reported concerns about the health of their pregnant partners and new-born infants as the major reason for smoking cessation. The participants expressed a willingness to change their behaviour, including smoking cessation, after realising the negative effects of SHS exposure on the health of pregnant women, foetuses and new-borns.

*‘They [pregnant wife and foetus] are very important to me now, you know [laughs], I can do everything for their health. So, I quit smoking immediately as requested by my family members after I knew my wife got pregnant.’ (Informant 015)*

In addition, some participants stated that by mentioning their wife’s pregnancy, they had been forgiven for refusing cigarettes shared by friends or colleagues. This provided them an opportunity to quit smoking or maintain cessation.

*‘When my friend handed the cigarettes to me, I often told them that I was preparing for pregnancy or my wife was pregnant and didn’t like the smell of smoking. Then they generally expressed their understanding of my situation and forgave my cigarette-refusing behaviour.’ (Informant 009)*

#### The role of being a father

The desire to act as what respondents considered to be a good father acted as a strong motivator for smoking cessation among expectant fathers, especially those who were new to fatherhood. Some participants reported that smoking cessation was not only beneficial to the health of their wives and children but also improved their image and enabled them to be good role models for their future children.

*‘I cannot smoke after my child is born. I don’t want my child to develop bad habits in the future because of my influence. Teach by example, you know. I should act as a good example.’ (Informant 018)*

#### Encouragement to quit from family members

Family members who encouraged and recognised that smoking cessation was beneficial for the family appeared to promote the ability of expectant fathers to achieve and maintain smoking cessation. This encouragement and recognition increased their sense of control of and participation in their partner’s pregnancy. However, this theme was only reported by participants who had taken actions to quit or reduce smoking.

*‘My whole family was very happy after I quit smoking during the pregnancy preparation period. I have maintained abstinence from smoking until now. I couldn’t help her (wife) with pregnancy. But I quit smoking for her and my baby, rather than just provide a sperm [laugh]. I feel proud of it.’ (Informant 021)*

### Theme 3. Perceived barriers to smoking cessation

Their partners’ pregnancies provided a good opportunity for expectant fathers to quit smoking. Eleven participants who attempted to quit were relapsed or failed to quit. Three perceived barriers were documented, including withdrawal symptoms or cigarette cravings, absence of smoking cessation support, and increased stress.

#### Withdrawal symptoms or cigarette cravings

Many participants claimed they failed to quit smoking because of the withdrawal symptoms. Cigarette cravings were frequently reported. Some participants reported difficulty concentrating when they attempted to quit smoking. All participants mentioned this barrier and expressed that they did not know how to deal with these symptoms during smoking cessation.

*‘I tried to quit before but failed. I don’t know what I should do for the cigarette cravings.’ (Informant 004)*

#### Absence of smoking cessation support

Some participants expressed a low level of willingness to seek help with smoking cessation. The participants reported that smoking cessation was a minor, personal issue, and therefore seeking help from official services was unnecessary. In some cases, participants attributed their failure to quit to a lack of personal determination. They believed that they could quit whenever they chose.

*‘There is no need to visit a doctor for something like quitting smoking. I can do it myself. I can quit any time when I want.’ (Informant 19)*

Several participants said they were not aware of smoking cessation services. Others highlighted an absence of pre-pregnancy or prenatal education on smoking cessation because they were unaware of the existence of the education programmes intended to help with that challenge or of their need to attend such programmes. Some explained that they had not even been allowed to enter the waiting area in the obstetrics and gynaecology clinic and consequently had received no education or support. Still others reported that the general smoking cessation clinics only operated on 1 or 2 days per week, and thus their schedules made it difficult to visit healthcare professionals for assistance with smoking cessation.

*‘There was a sign that said “women only” on the wall of the visiting room at the obstetrics and gynaecology clinic, so I waited for my wife outside each time; no one asked me to enter to receive education with my wife’. (Informant 016)*

#### Increasing stress

The participants stated that they felt stressed. Compared to the situation before the partner’s pregnancy, most participants reported a decreased family income due to the partner’s reduced workload or maternity leave. This decrease in the household economic situation pressured the expectant father to increase his workload, which increased the stress from both the household economic situation and the workplace. Some participants reported that it was difficult to give up smoking because they did not know other coping strategies for pressure and stress. Participants who had previously used smoking cessation services reported that the counselling was too shallow to assist them with cessation. Most participants claimed that smoking cessation clinics only provided nicotine patches, with minimal counselling or advice, and did not provide adequate support to help them cope with psychological issues.

*‘My wife stayed at home after she got pregnant. I’m working three jobs because the costs will increase much more after the baby is born. Smoking helps me feel relaxed when I’m tired and stressed.’ (Informant 022)*

## Discussion

This study investigated the perceptions, behaviours and attitudes related to smoking among expectant fathers. The findings may increase the awareness of this under-researched area and provide information to support the development of effective interventions to promote smoking cessation among expectant fathers.

This study determined that misconceptions about the effects of smoking and SHS led to the resumption of smoking. However, the observed associations between the perception of health and smoking or SHS revealed that expectant fathers lacked accurate knowledge about the hazards of SHS. A previous study suggested that more specific health information could help to increase this knowledge and correct misconceptions about smoking hazards [26]. Information about how smoking and SHS specifically influence the health of pregnant women and children may help to enhance men’s concerns and motivation to quit. Hence, interventions that deliver information may be more effective among expectant fathers. Still, the situation involving subsequent pregnancies may be more complex, as the discrepancy between the smoking cessation advice provided by healthcare professionals and the fathers’ previous experience with healthy babies born into smoking families reduced their perceived need to quit [27]. This information explained the association between subsequent fatherhood and a low cessation rate, which was observed in a previous quantitative study [15]. This finding emphasises the promotion of education to address the misconceptions in this population and the need for further studies to explore the factors that motivate subsequent fathers to quit smoking.

Consistent with data from a Chinese population study in 2016, the participants, particularly those who were younger, reported that they were not concerned about the impact of smoking on their health because this would develop slowly [28]. Our findings indicated that concerns about the health of the pregnant partners and babies, and the role of being a father were reported by expectant fathers as the strongest motivations for smoking cessation at this life stage. During this special stage, expectant fathers may be prepared to make significant behavioural changes, particularly in terms of taking more responsibility for their actions [29]. Hence, they may be open to appeals to quit smoking. Compared to personal smoking-related health problems in the future, the hazards to the health of pregnant women and children appear to be more concrete and proximal to the lives of expectant fathers. In addition, the latter has a greater preventive significance because having these hazards occur before personal health problems would motivate a male smoker to quit. This suggests that appropriate interventions that focus on these concerns and highlight the benefits of quitting could encourage these men to consider smoking cessation and a healthy lifestyle [12].

As noted above, smoking has long played an important social function in Chinese culture. In a previous study, smokers who planned to quit were often challenged by a custom wherein the refusal of offered cigarettes is considered impolite [7]. However, the findings in theme three revealed that the partner’s pregnancy provided a good excuse for refusing tobacco use and thus facilitates smoking cessation success. Furthermore, the encouragement provided by family members and their recognition of efforts to quit smoking were also identified as a facilitator by expectant fathers. Physiologically, the contributions of expectant fathers during pregnancy are limited, and they may feel excluded [30]. Accordingly, the encouragement and recognition of smoking cessation as a beneficial behaviour by other family members would increase the expectant father’s sense of control and participation during the partner’s pregnancy, which could support the maintenance of cessation. Therefore, family support should be considered in relapse prevention strategies for expectant fathers.

Our findings also identified several barriers to quitting smoking among expectant fathers. In general, nicotine withdrawal symptoms appear soon after tobacco abstinence and can last for several weeks [31]. This is consistent with previous literature stating that smokers who experience more severe withdrawal symptoms are less likely to achieve long-term abstinence [32]. Therefore, additional smoking cessation support should be provided to equip smoking expectant fathers with the skills to overcome withdrawal symptoms. However, consistent with the gendered expectations of masculinity in the Chinese context, the expectant fathers expressed an attitude of resistance and were more unwilling to seek help for smoking cessation than were women [33]. Accordingly, healthcare professionals should take more initiative in approaching this population to promote smoking cessation. By 2019, more than 350 smoking cessation services had been established in China [34]. However, the expectant fathers in our study reported an unawareness of these services and found it difficult to accept support for smoking cessation. More publicity and referral approaches should be developed to enhance the use of smoking cessation services, and the settings should be improved to improve access by expectant fathers. In addition, people who lack the skills to handle negative emotions, such as stress related to economic contraction, would wrongly use smoking as a coping mechanism [35]. In this scenario, the excitatory effects induced by nicotine could help them to release the stress directly [36]. Furthermore, a previous cohort study reported that when compared to women, men were more likely to exhibit increased smoking behaviours in response to economic stress [37]. A study found that smokers have lower long-term income and earnings [38]; the increasing cost for the baby may aggravate the expectant fathers’ economic stress, which further increased their need to smoke. Although 96% of smoking cessation clinics are reported to provide counselling [34], the support available to address psychological issues may not be adequate to meet the needs of smokers. This suggests that counselling should aim to provide more skills to help smokers cope with emotional problems. Per the World Health Organisation’s recommendation, smoking cessation services should be made more available to smoking expectant fathers and should provide support to equip them with the skills to overcome withdrawal symptoms and cope with psychological issues to enhance smoking cessation [39].

### Strengths and limitations

The strength of this study is the use of the qualitative approach to collect in-depth information directly from expectant fathers, and it thus addresses gaps in the existing literature. Another strength is the purposive sampling approach used in this study, which was selected according to the smoking cessation status and related factors with the intention to include all possible scenarios for this population. This approach allows for a rich, in-depth understanding of the phenomenon of smoking cessation among expectant fathers.

However, this study was limited because we did not approach smoking expectant fathers with smoking partners at the clinics, as the prevalence of smoking among women was relatively low in China (2.4%) [3]. A further study should be conducted to enrich knowledge in this area.

### Implications for practice

This study provides information of important relevance to future research and practice. First, pregnancy offers a valuable opportunity for expectant fathers to change their behaviours and lead healthier lives. Considering the increasing family role responsibilities for expectant fathers, smoking cessation interventions applied at this stage may yield better effects than those imposed at other times. Second, concerns about the health of pregnant women and infants were identified as the main facilitators of smoking cessation, while a lack of specific and accurate knowledge about tobacco use served as a barrier to smoking cessation among Chinese expectant fathers. This finding suggests that interventions intended to promote smoking cessation in this population should focus on emphasizing hazards of smoking to maternal and neonatal health, providing greater health-related knowledge and correcting common misconceptions. Third, the tendency of the expectant fathers towards masculinity appeared to lead to a reluctance to seek help from smoking cessation services. Hence, healthcare professionals should actively approach this population to enhance smoking cessation. Fourth, further interventions should consider including family support, which may potentially increase the smoking cessation rate in this population. Finally, given the increased pressure and the difficulties in dealing with withdrawal symptoms by expectant fathers, appropriate interventions should be developed and evaluated with the aim to help them better cope with the stress during the transition to fatherhood. Additionally, more strategies should be introduced to help expectant fathers overcome withdrawal symptoms.

## Conclusion

This study provides a comprehensive understanding of the perceptions of, behaviours towards, and attitudes about smoking amongst Chinese expectant fathers. Despite the limitations of our study regarding unreached smoking expectant fathers with smoking partners, the information above could guide healthcare professionals and policymakers to combine the educational information about the hazards of SHS for maternal and neonatal health and smoking cessation assistance for expectant fathers with policy initiatives and other types of incentives and programmes aimed at enhancing smoking cessation amongst this population. To enrich knowledge in this area, further research can explore the reasons explaining the smoking behaviours of expectant fathers’ partners who smoke during pregnancy.

## Supplementary Information

**Additional file 1: Supplementary 1.** Information of research team.

**Additional file 2: Supplementary 2.** Interview guideline.

## Data Availability

The datasets used and/or analysed during the current study are available from the corresponding author, subject to approval from the ethics committee that approved the original study.

## Abbreviations

SHS: Secondhand smoke
COREQ: Consolidated Criteria for Reporting Qualitative Research
