# Supplementary material for: Perceptions, behaviours and attitudes towards smoking held by the male partners of Chinese pregnant women: a qualitative study
Source: BMC Public Health. 2021 Oct 20;21:1901. doi: 10.1186/s12889-021-11966-4 (PMC8527705; doi:10.1186/s12889-021-11966-4)
Supplement: Supplementary file 1 — Additional file 1: Supplementary 1. Information of research team. [file 12889_2021_11966_MOESM1_ESM.docx]

Supplementary 1. Information of research team

| Member | Gender | Occupation | Credentials | Qualification | Length of experience | Others |
| --- | --- | --- | --- | --- | --- | --- |
| WX | F | Associate professor | PhD, MPhil | Registered nurse | 5 years | Guideline developer, Coder |
| WHCL | M | Associate professor | PhD, MPhil | Registered nurse | 15 years | Guideline reviewer |
| KYH | F | Assistant professor | PhD, MPhil | Registered nurse | 8years | Guideline reviewer |
| PS | F | Research professor | PhD, MPhil |  | 5 years | Coder |
| YHL | F | Post-doctoral Fellow | PhD, MPhil | Registered nurse | 4 years | Interviewer |
| TL | F | PhD candidate | MPhil | Registered nurse | 3years | Interviewer |
| LLKH | F | Post-doctoral Fellow | PhD, MPhil | Registered nurse | 4 years | Observer |
| TAC | F | Post-doctoral Fellow | PhD, MPhil | Registered nurse | 4 years | Audit recorder |
