# Supplementary material for: Perceptions, behaviours and attitudes towards smoking held by the male partners of Chinese pregnant women: a qualitative study
Source: BMC Public Health. 2021 Oct 20;21:1901. doi: 10.1186/s12889-021-11966-4 (PMC8527705; doi:10.1186/s12889-021-11966-4)
Supplement: Supplementary file 2 — Additional file 2: Supplementary 2. Interview guideline. [file 12889_2021_11966_MOESM2_ESM.docx]

Supplementary 2. Interview guideline

| 1. Can you talk about the change of smoking behaviors before and after your partner got pregnant? Why? 2. Can you talk more about your experience of such behavior? 3. Can you talk about how do you think about the tobacco use? 4. What do you know about the influence of the tobacco use? 5. What factors do you feel may influence your behavior change? 6. Can you talk about the barriers you experienced during the smoking cessation? 7. How do you think about the behavior of seek help for quitting smoking? 8. Have you ever seek help for quitting smoking? Can you describe your experience? 9. Is there any other information you want to talk about the tobacco use among your partner’s pregnancy? |
| --- |
